# Flexible microscale tactile display with liquid-to-gas phase-change actuator array

**DOI:** 10.1038/s41378-026-01288-z

**Published:** 2026-05-19

**Authors:** Sangjun Sim, Kyubin Bae, Kyuhyun Hwang, Seunghwan Koh, Jongbaeg Kim

**Affiliations:** 1 School of Mechanical Engineering, Yonsei University, Seoul, Republic of Korea; 2 Walker Department of Mechanical Engineering, The University of Texas at Austin, Austin, TX USA

**Keywords:** Electrical and electronic engineering, Chemistry

## Abstract

Flexible tactile displays have gained significant attention for their ability to deliver tactile feedback to users, complementing widely used visual displays and auditory output devices. However, previously reported tactile displays have limited spatial resolutions because they are mostly confined to centimeter-scale sizes, restricting the provision of sophisticated tactile feedback. We developed a microscale flexible actuator using water as a liquid phase-change material (PCM), which exhibits a large volumetric expansion upon phase transition from liquid to vapor. The actuator consisted of a flexible microheater, PCM, and stretchable elastomer membrane. A scalable batch fabrication process was used to form PCM chambers localized within the heater regions. Microscale PCM actuators have a thickness of less than 200 μm, making them easy to attach to curved surfaces. The device achieves a response time of 600 ms and a displacement of 580 μm and operates at a low power consumption of 300 mW. Integrating this tactile display with a commercial virtual-reality headset for visual feedback enables immersive multisensory information delivery to users.

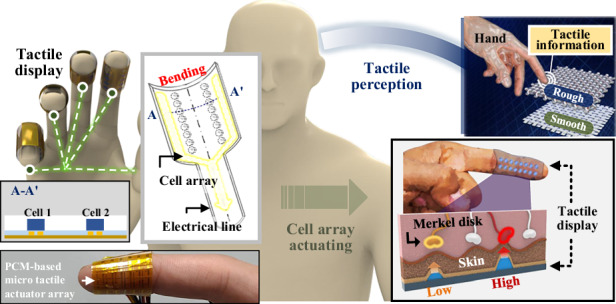

## Introduction

User-interactive real-time feedback technologies, such as virtual reality (VR), augmented reality, and human–machine interfaces, have advanced along with the development of devices that transmit information by stimulating human senses, including vision^1–4^, hearing^5,6^, and touch^7,8^. In particular, haptic interfaces, which provide kinesthetic or cutaneous feedback, have garnered attention for enhancing the immersive experience of users, bridging the gap between the real and virtual worlds^9–12^. Wearable robotic gloves capable of controlling wire tension have been developed^13–15^. These devices effectively convey information about the shape or stiffness of objects to provide kinesthetic feedback to users. Moreover, cutaneous feedback methods have been developed using devices that apply vibration^16^, temperature^17^, and electrical stimulation^18^ to the skin. These devices enable the transmission of more sophisticated tactile information, which was previously limited to kinesthetic feedback.

The human fingertip has the highest density of tactile receptors among all skin areas, with over 200 receptors cm^–2^ distributed across its surface^19–21^. Several researchers have endeavored to transmit the surface information of objects through the mechanical stimulation of the skin on the finger^22–24^. Despite extensive efforts, realizing realistic tactile feedback remains a significant challenge due to the technical difficulty of implementing actuator arrays that simultaneously achieve large displacement and high spatial resolution for effective mechanoreceptor stimulation. To address these stringent requirements, various mechanisms for tactile display have been proposed, including electromagnetic^25,26^, piezoelectric^27^, electrostatic^12^, and pneumatic actuators^28^. For instance, Kim et al.^29^ reported a piezoelectric-actuated tactile display with a high spatial resolution of 1.8 mm and a fast driving time of less than 1 ms. However, the driving displacement of the device is limited to 1 μm, restricting its ability to stimulate mechanoreceptors at varying pressure levels^30^. Moreover, the rigidity of the individual cell materials makes it difficult for the device to adhere closely to the fingertip. Shea et al.^12^ developed a flexible tactile display using an electrostatic actuator with a fast response time of less than 10 ms. However, there was a mismatch between the large size of the actuator cells and the size and distribution density of the tactile receptors, limiting the ability of the system to convey detailed tactile information.

Phase-change material (PCM)-based actuators have recently gained attention for tactile displays because of their higher output power compared with other actuation mechanisms^31^. In addition, liquid-to-gas PCMs are encapsulated in stretchable polymer pouches, providing mechanical flexibility to the devices. Several researchers demonstrated tactile displays capable of generating large forces by utilizing the vaporization of liquid-to-gas PCMs. However, these displays suffer from slow driving speeds (>10 s) and high energy consumption (>10 W) primarily because of their size (>1 cm)^32,33^. Moreover, the practicality of delivering sophisticated tactile information is limited by a lack of array configurations.

In this study, a flexible tactile display was developed using a microscale liquid-to-gas phase-change actuator. Phase-change expansion describes the volumetric expansion of the sealed chamber arising from the liquid-to-gas transition of the working fluid during heating. The display was composed of microheaters, water as the PCM, polymer substrates, and membranes, resulting in a fully flexible device. In a previous study, we developed a flexible PCM actuator by placing water droplets on a microheater^34^. However, the low uniformity of the water droplets caused deviations between the cells. To address the non-uniformity of water droplets, we propose a new fabrication method for liquid PCM chambers that enables consistent PCM volume across the array. Ice particles were formed on the substrate using a sublimation setup, and frozen water was spin-coated and passivated with an Ecoflex layer. This approach enables the formation of thin membranes over an array of the liquid PCM in a batch process. The fabricated display had a cell diameter of 500 μm, intentionally designed to be smaller than the ≈1 mm spatial resolution of Merkel disk receptors, enabling localized and repeatable stimulation of these mechanoreceptors. The PCM actuators can achieve a maximum actuation displacement of 580 μm, which is comparable to or exceeds the vertical indentation required for perceivable static pressure on human skin^19,35^, while operating with a fast response time of 370 ms. This response time is substantially faster than previously reported centimeter-scale liquid-to-gas PCM actuators, which typically require several seconds for actuation. These advantages allow us to successfully demonstrate a tactile display capable of generating and interacting with sophisticated touch sensations. Moreover, we demonstrate the ability of the device to deliver detailed tactile information, such as the sensation of a ladybug crawling on the skin.

## Results and discussion

### Operating mechanism of a microscale tactile display

**Fig. 1:**
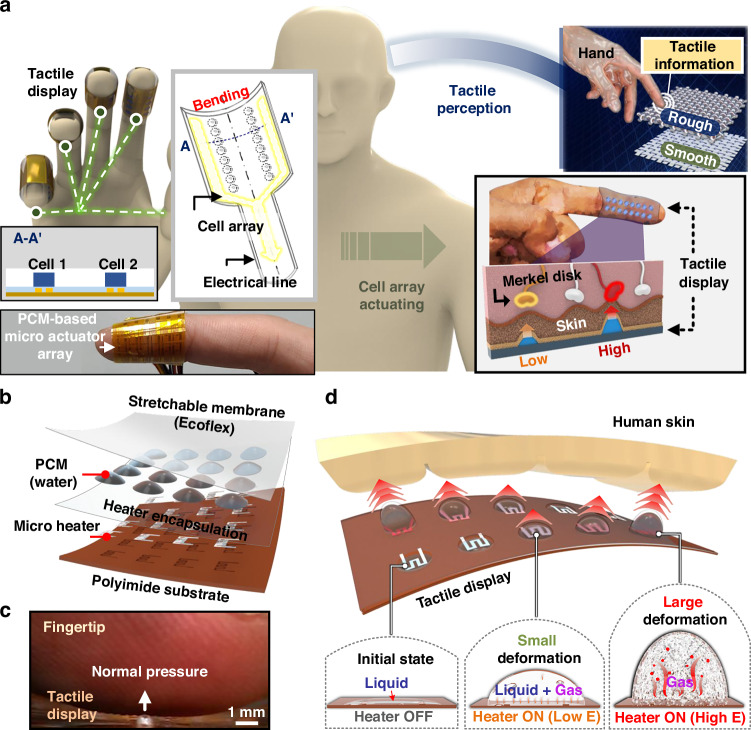
**Schematic of the utilization of a flexible microscale tactile display to deliver tactile information.** **a** Applications of tactile displays. The tactile display can be worn on the finger and can be easily deformed to fit the curved shape without any issues. Subsequently, the tactile display is capable of inducing continuous displacement in the normal direction, effectively both weakly and strongly stimulating the Merkel disks. This allows users to identify the surface properties of objects, such as roughness. **b** Detailed illustration depicting the individual components of a fully flexible tactile display in an exploded view. **c** Optical image of a tactile display and finger. The PCM actuator cell exerts pressure on the finger in the normal direction when driven. **d** Schematic depicting the continuous driving of cells in each tactile display. The vaporization amount of the liquid PCM can be continuously adjusted based on the energy applied to the heater, allowing the actuator to be driven continuously in the normal direction

Figure 1a shows a schematic of the flexible tactile display based on the liquid-to-gas PCM actuator array. The microheater and liquid PCM, which constituted the components of the PCM actuator, were constructed on a flexible substrate and covered with an easily deformable polymer membrane. Although PCM actuators are generally associated with relatively slow response speeds due to the kinetics of phase transition, the present device mitigates this limitation through aggressive miniaturization. By scaling down the active volume and thermal mass, both the heating and cooling times are substantially reduced, enabling a sub-second actuation response (~370 ms) in the microscale PCM actuator (Movie S1 in the Supplementary information). These materials provide high flexibility to the device, enabling it to deliver sophisticated tactile sensations by wrapping it around the user’s fingertips. Our device, consisting of an array of cells smaller than 1 mm in diameter, can apply a variable force by adjusting the actuation displacement and delicately stimulating the tactile receptors in the skin. Among these receptors, the Merkel disk, which detects static pressure with a spatial resolution of approximately 1 mm^35^, has been particularly difficult to stimulate using conventional flexible devices due to limitations in actuator miniaturization. To overcome this challenge, our device was designed with a sub-millimeter cell size, allowing localized and repeatable stimulation of Merkel receptors. By stimulating these tactile receptors with a high spatial resolution, precise surface information, such as an object’s roughness, can be conveyed.

Figure 1b shows a schematic of the PCM actuator array. It was composed of a thin polyimide (PI) substrate with mechanical flexibility, and a patterned Au layer was used as the microheater array. Water droplets served as the PCM, utilizing their 0–100 °C phase-change range to enable freezing during fabrication and vaporization during actuation. A PI passivation layer was deposited on the metal microheater to enhance its thermal and chemical stability, thereby minimizing oxidation and degradation of the metal layer during repeated heating cycles^36,37^. The Ecoflex membrane, which is a stretchable elastomer, expanded as the PCM vaporized. As shown in Fig. 1c, the PCM actuator of the tactile display was in contact with the fingertips. The PI substrate has a Young’s modulus of 2.48 GPa^38^, whereas the Ecoflex membrane exhibits a Young’s modulus of 166 kPa^39^, indicating an elastic modulus difference of over 10^4^ between the two materials. The pressure generated as the PCM vaporizes primarily deforms the Ecoflex membrane, applying an upward normal force toward the skin in contact.

Figure 1d illustrates the actuation mechanism of the PCM actuator, to which various displacements can be applied. In the initial state, no power was applied to the heater; therefore, the PCM remained in the liquid state. In this step, a flat surface was formed, and no pressure was applied to the skin of the user. When power was applied to the heater, heat was transferred to the liquid PCM, causing some of it to vaporize. As the PCM vaporized in the chamber, the volume expanded, and the Ecoflex membrane deformed upward accordingly. This deformation allowed pressure to be transmitted by contact with the user’s skin. When the PCM recondensed into a liquid and returned to its original volume, the pressure dissipated, returning the system to its initial state (i.e., flat surface). Applying higher power vaporized more of the PCM, inducing greater membrane deformation and increasing pressure transmission. The proposed tactile display uses a PCM to generate continuously tunable displacement, enabling the modulation of indentation depth above the just-noticeable-difference (JND) threshold of human skin. With a peak displacement of 580 µm, the device spans the perceptual range from clearly detectable indentation (above the skin JND of ≈10–100 µm, depending on contact area^40,41^) to salient static pressure cues (>300 µm^42^). Combined with a sub-millimeter cell array, it enables (i) intensity-graded pressure through amplitude control, (ii) spatial patterns (edges, points, and rows) for texture-like cues at the fingertip^43^, and (iii) low-frequency temporal sequences for dynamic tactile rendering.

### Fabrication of a tactile display using PCM actuator arrays

**Fig. 2:**
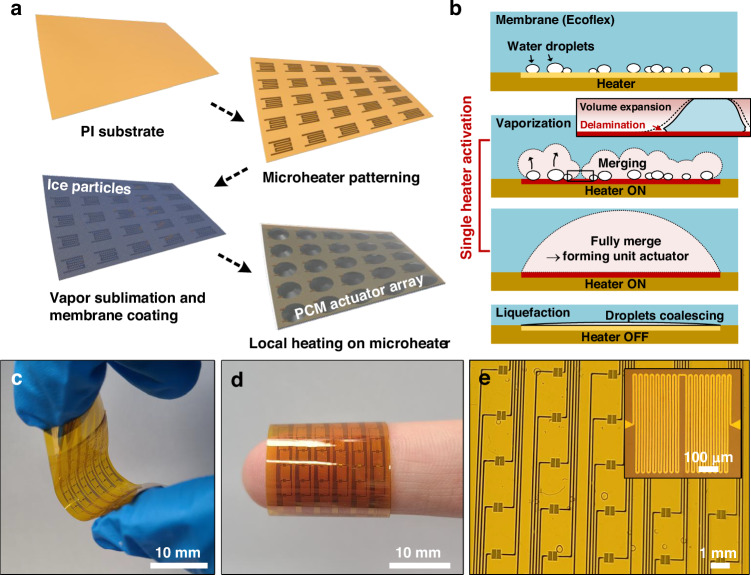
**Fabrication process of flexible PCM actuator arrays.** **a** Schematic of the fabrication process of a PCM actuator array using a PI substrate and microheater patterning. The process includes vapor sublimation and membrane coating after ice particle deposition, followed by local heating to form the actuator array. **b** Schematic of the working mechanism of a single PCM actuator. Upon heating, water droplets on the Ecoflex membrane vaporize and expand, causing membrane delamination. The droplets merge and form a unit actuator, which deactivates and liquefies upon cooling. **c** Photograph of a flexible microheater array on a PI substrate, demonstrating mechanical flexibility. **d** Close-up image showing the microheater array wrapped around a finger, illustrating its conformal properties. **e** Optical microscopy image showing a magnified view of the microheater array, with the inset displaying the detailed heater structure at the microscale (scale bar: 100 μm)

Figure 2a shows a schematic of the fabrication process of the PCM actuator arrays. We patterned an Au microheater on a PI substrate. Subsequently, ice was formed on the surface using custom-made sublimation equipment (Fig. S1 of Supplementary information). In this state, the Ecoflex as a membrane was spin-coated. After curing the Ecoflex, a liquid PCM chamber was created only on the heater through local heating (the detailed fabrication processes are described in the Experimental Section and Fig. S2 of the Supplementary information). Figure 2b illustrates the technique for forming individual PCM chambers in actuator arrays for the first time. When Ecoflex was spin-coated and cured on the ice formed on the heater, individual water droplets were trapped in the chamber throughout the area. When the heaters were locally heated, only the water droplets directly above the heater were vaporized. In contrast, the water droplets located between the heaters, which were not sufficiently heated, remained in the liquid state. The expanded water vapor separated from the Ecoflex and substrate and combined with other droplets to form a chamber (the high-speed camera image of the chamber formation process is included in Fig. S3 in the Supplementary information). As heat was generated only in the heater area during device operation, a chamber was formed only in that area. When the heater was turned off after formation, the vapor condensed into a liquid, completing the PCM actuator array. It is noteworthy that a uniform cell can be formed in the heater area without requiring a separate chamber layer or any water patterning process.

Figure 2c–e shows the optical images of the PCM actuator array. As shown in Fig. 2c, the fabricated PCM actuator array is thin and flexible. With these characteristics, the device was wrapped around the finger for use as a tactile display (Fig. 2d). It can also be fabricated and utilized at various scales (Figs. S4 and S5 in the Supplementary information). For example, a high-density prototype with 25 individually addressable cells integrated within a 3 mm × 3 mm area was successfully fabricated, demonstrating that the phase-change actuation mechanism itself does not impose a fundamental limitation on miniaturization. Figure 2e shows an enlarged image of the heater arrays of the PCM actuator. An analysis of the heating characteristics of the fabricated heater is shown in Fig. S6 in the Supplementary information. A stable heating performance was observed even at temperatures above 100 °C, which vaporized the liquid water. As confirmed by thermal imaging (Fig. S7), the high-temperature region above 100 °C is confined within ~1 mm around the heater, while the temperature in the surrounding and intermediate regions remains near 30 °C, close to ambient conditions. With a cell pitch of 3 mm, thermal crosstalk between neighboring actuators is therefore negligible.

### Characterization of the tactile display

**Fig. 3:**
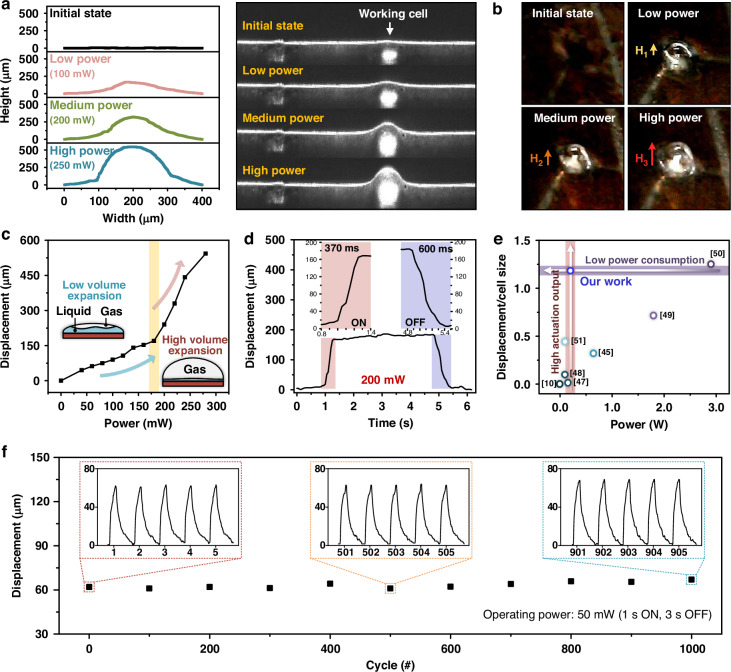
**Actuation characteristics of the PCM-based actuator under constant-voltage driving mode.** **a** Cross-sectional profiles and corresponding infrared images of the working cell under different power levels (100, 200, and 250 mW). The height increases with the power input, indicating the expansion of the actuator. **b** Infrared thermal images showing the displacement of the working cell at different power levels (low, medium, and high) as marked by H_1_, H_2_, and H_3_, respectively, compared with the initial state. **c** Displacement of the actuator as a function of the power input. The inset illustrates the transition from low volume expansion (liquid-to-gas phase) to high volume expansion (gas phase). **d** Time-resolved displacement response of the actuator under an input power of 200 mW, demonstrating fast actuation (370 ms) and recovery (600 ms). **e** Comparison of displacement efficiency (displacement per cell size) as a function of power consumption with previous works. The proposed actuator shows high efficiency with low power consumption. **f** Long-term actuation stability test over 1000 cycles under repeated actuation at 50 mW (1 s on/3 s off). The insets show the detailed displacement response during the initial, middle, and final cycles, confirming the reliability of the actuator

We measured the surface profiles of the flexible PCM actuator at the power levels of 100, 200, and 250 mW (low, medium, and high powers) using a laser displacement sensor, which also provided side-view optical images of the actuator as shown in Fig. 3a (detailed information on the measurement setup is shown in Fig. S8 in the Supplementary information). A PCM actuator with an integrated microheater can control the amount of water vaporization, liquid PCM, and volume within the chamber, depending on the driving power of the heater. In other words, the actuating distance is adjusted by changing the power. As shown in Fig. 3a, b, when no power was applied in the initial state, no displacement occurred (i.e., a flat surface). As the driving power increased, the displacement increased from 0 to 500 µm. Owing to the localized heating of the microheater, selective actuation of individual cells was observed. Figure 3c shows the displacement of the PCM actuator at different driving powers. As the power of the microheater increased from 0 to 300 mW, the actuation displacement was measured to range from 0 to 580 µm. The slope of the displacement–power graph can be divided into two distinct regimes, corresponding to different dominant thermal processes. To quantitatively capture this behavior, the full power–displacement dataset was analyzed using a continuous piecewise-linear regression model, which enables an objective identification of the transition point between the two regimes. The threshold power *P*_*b*_ marking the onset of vaporization-dominated expansion was estimated directly from the experimental data by minimizing the global sum of squared errors and is found to be approximately 180–190 mW (Fig. S9a). In the first region (*P* < *P*_*b*_), heat was consumed to increase the liquid PCM temperature, resulting in a displacement increase rate of 0.94 µm/mW. When power is applied, the heat generated by the heater increases the water temperature, causing only a portion of the liquid to vaporize. At higher power (*P* > *P*_*b*_), the heater temperature reaches 100 °C, vaporizing the remaining water and resulting in a substantial expansion of the chamber volume. In the second region, the displacement increase rate rises to 2.9 µm/mW, and most of the heat energy is used to vaporize the liquid, leading to greater volume expansion. This transition between the two regions is reflected in the change in slope of the power–displacement curve in Fig. 3c, indicating a shift from sensible heat dominated heating to latent-heat-driven vaporization. The validity of this interpretation is further supported by the small residuals between the experimental data and the fitted model across most of the power range, indicating good agreement between the analytical framework and the measured response (Fig. S9b). A detailed analytical model describing the coupling between input power, vapor generation, internal pressure, and membrane deformation is provided in Supporting Note 1.

Figure 3d shows the transient response curves of the device. At 200 mW, the response and recovery times were 370 and 600 ms, respectively. Previously reported phase-change actuators exhibited slower response times, in the range of several seconds^32,44–46^. Owing to its small volume and heat capacity, our device operates rapidly in less than 1 s, achieving displacements of up to 200 μm. Figure 3e compares the driving power and operating displacement per unit size of our device with those of previously reported actuators^9,45,47–51^ (see Table S1 in the Supplementary information for detailed information). Our device operates with a low power consumption of 300 mW while achieving a driving displacement of 580 µm, which exceeds the unit cell diameter of 500 µm. Some studies have reported that an actuation power of less than 500 mW is possible^9,47,48,51^. However, these devices exhibit limited driving displacement per unit cell size. Compared with state-of-the-art actuators, which require a large footprint area, our device has a large actuation displacement and low power consumption. Furthermore, we compared the performance of recently reported PCM actuators and our device, as shown in Table S2 in the Supplementary information.

To further evaluate environmental robustness, the actuator performance was characterized under different humidity and temperature conditions. The device was operated at relative humidity levels of 25%, 45%, and 65% while maintaining identical driving power and timing, and no noticeable change in displacement amplitude or actuation profile was observed (Fig. S10a). In addition, the actuator was tested at ambient temperatures of 23 °C, 36 °C, and 50 °C under the same driving conditions. Although a slight increase in displacement was observed at elevated temperatures due to facilitated phase transition, the overall actuation behavior remained stable and repeatable (Fig. S10b). Furthermore, long-term storage stability was evaluated by remeasuring the same device after more than one year of storage at room temperature under ambient laboratory conditions. Under identical driving conditions (100 mW), the peak displacement decreased only slightly from 91 μm to 84 μm, corresponding to a 7.7% reduction, while the transient displacement profiles remained nearly unchanged (Fig. S11). This result indicates that the volume of the water-based PCM is well preserved within the sealed chamber, with no noticeable vaporization, leakage, or drying over time. In addition, although the Ecoflex membrane is cast on an ice substrate during fabrication, the curing process is completed at room temperature, ensuring stable film formation and complete curing, as supported by the preserved actuation performance and intact membrane morphology after long-term storage.

The device was repeatedly operated for 1000 cycles at a power of 50 mW to evaluate its driving displacement characteristics, as shown in Fig. 3f. The actuator exhibited stable and repeatable deformation behavior throughout the test, with displacement variation remaining within 9% during repeated liquid-to-gas phase transitions. In addition, optical microscopy images acquired before and after cycling confirmed that the cell boundary and dome geometry were well preserved, with no observable delamination or degradation at the Ecoflex–PI interface (Fig. S12). These results demonstrate the structural robustness and packaging stability of the actuator under prolonged operation.

Haptic feedback devices must generate a wide range of driving forces. This capability allows the realization of fine surface textures and the transmission of sophisticated tactile sensations. In this study, force output refers to the normal contact force generated at the actuator surface as a result of internal pressure induced by liquid–gas phase change. Fig. S13a shows the force measurement setup while the device was operating, indicating the driving force of the PCM actuator at various applied powers. The force gauge with a rubber tip, which has a similar Young’s modulus (≈100 kPa) to that of a human fingertip, was in contact with the deformable membrane of the actuator, allowing the measured force to reflect the effective force transmitted through the soft membrane under realistic skin-like contact conditions. When power was applied to the actuator, the liquid PCM vaporized, expanded in volume, and stretched the membrane. The repulsive force exerted on the rubber tip was measured during this process. Fig. S13b shows an optical image captured during the operation (an operating video is included in Movie S2 in the Supplementary information). To imitate the close contact between the skin and the device, a preload of 1 N was applied before device actuation. Fig. S14 shows the measurement results for repeated operation over 60 cycles at a driving power of 50 mW. The slight drift observed during cycling is attributed to thermally induced pressure fluctuations and membrane relaxation associated with repeated vaporization and condensation of the PCM. Importantly, the magnitude of this drift remains below the reported JND for fingertip-based quasi-static tactile perception (≈10–15%) and is therefore perceptually indistinguishable to users, without affecting practical actuation performance^22,52,53^. Fig. S15 depicts the driving forces at various power levels. As the power increased from 40 to 180 mW, the actuation force increased from 15 to 60 mN. This force range exceeds the reported perceptual threshold for normal-force detection at the human fingertip^12^, indicating that the generated force is appropriate for effective tactile feedback in wearable haptic applications.

We fabricated flexible PCM actuator arrays with arranged up to 6 × 6 cells using the batch fabrication process shown in Figs. 2a and S2 in the Supplementary information. The actuator, composed of a PI substrate and an Ecoflex substrate, exhibited mechanical flexibility, enabling conformal contact with curved surfaces, such as a fingertip. In addition, the batch-fabricated device ensured little variation among the fabricated 36 heaters, resulting in low resistance variation and consistent heating characteristics, as shown in Fig. S2 of the Supplementary information.

**Fig. 4:**
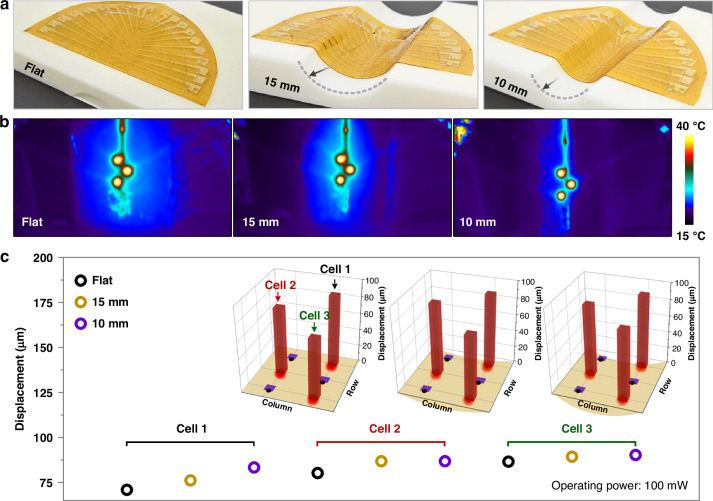
**Performance of a flexible PCM actuator array under different bending radii.** **a** Photographs of the flexible actuator in flat, 15 mm, and 10 mm bending states, showing its adaptability to various curvature conditions. **b** Infrared thermal images of the actuator in different bending states (flat, 15 mm, and 10 mm), highlighting the uniform heat distribution across the array even when bent. **c** 3D bar graphs depicting the displacement of three individual cells (Cell 1, Cell 2, and Cell 3) measured under constant-voltage driving at an input power of 100 mW under different bending radii (flat, 15 mm, and 10 mm), demonstrating consistent actuation performance regardless of the degree of bending

Figure 4a shows the images of a flexible tactile display consisting of PCM actuator arrays attached to flat and curved surfaces with the radii of curvature of 15 and 10 mm, respectively, resembling the curvature of a finger^54^. The device conformed well to not only flat but also curved surfaces. Three cells were simultaneously operated while being attached to a curved surface, and the surface temperature of the actuator was measured using an infrared camera (Fig. 4b), indicating that the measured surface temperature stays at a level suitable for safe skin contact. The heating remained uniform across the three activated cells, even when attached to a curved surface, similar to the flat state. Notably, the vapor within the chamber and polymer membrane possessed high thermal resistance, preventing the surface temperature of the heater from being directly transferred to the user. The heater is not in direct contact with the user, and heat transfer is mitigated by the intervening vapor layer and the Ecoflex membrane, which has intrinsically low thermal conductivity. Although the surface temperature of the cells reached ~40 °C, which is sufficient to convey a slight sense of warmth, it remained significantly lower than the operating temperature of the heater, which exceeded 100 °C to vaporize water. Figure 4c shows the measured displacement when a specific cell was actuated on flat and curved surfaces. The pitch between adjacent cells was 5 mm, the displacements of the actuated and adjacent cells (i.e., non-actuated) were measured, confirming that the displacement occurred exclusively in the actuated cell. Under the same driving power, a small deviation of 6.2% was observed when comparing the curved and flat surfaces. These results demonstrate that the device can operate stably, even when attached to surfaces with varying curvatures.

### Application for the wearable tactile interface

VR is gaining significant attention in various fields, such as healthcare and entertainment industries^55,56^. Accordingly, devices that transmit information to users in immersive environments have been developed. Recently, technologies aimed at enhancing virtual experiences through tactile feedback have gained attention, with the objective of providing users with a more immersive and realistic experience by conveying a sense of touch^7,8^. Tactile feedback denotes the perceptible mechanical stimulus delivered to the user’s skin by the actuator displacement and contact force.

**Fig. 5:**
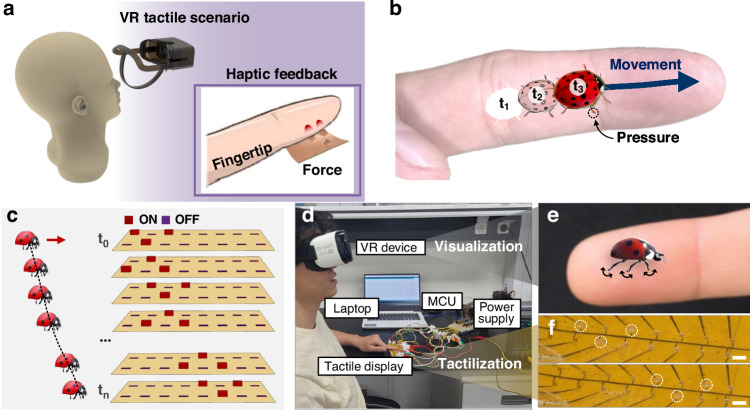
**Demonstration of a VR-based tactile feedback system utilizing a flexible microscale tactile display for haptic interaction.** **a** Schematic of a VR tactile feedback scenario where a flexible actuator delivers force to the user’s fingertip for haptic sensation. **b** Illustration of tactile feedback delivered by the system, simulating the movement and contact sensation of a ladybug walking across the user’s fingertip. **c** Schematic of actuator array operation corresponding to the movement of the ladybug across the fingertip, where individual actuators are turned on and off to simulate the tactile sensation in real time (ON/OFF duration: 450/150 ms). **d** Experimental setup showing the integration of the VR device with the tactile display system, including a laptop, an MCU, and a power supply (driving conditions: actuation power = 200 mW, displacement ≈ 150 µm). **e** Close-up image of a ladybug on a fingertip, illustrating the tactile simulation of its movement. **f** Microscopic image of the actuator array used for tactile feedback (scale bars: 1 mm)

Hence, we developed a multimodal VR interface capable of simultaneously delivering visual and tactile information to the users (Fig. 5a). The device consisted of a commercial VR headset for visual output and a flexible tactile display that conveys tactile sensations to the fingertips. The PCM actuator arrays of the tactile display were synchronized with the visuals displayed on the headset to provide coordinated tactile feedback. As shown in Fig. 5b, we demonstrated that computer graphics of virtual ladybug, created using video-editing software, crawls on a user’s finger. Our device was composed of PCM cells of diameter 500 µm, arranged in a 2 × 12 configuration, allowing us to recreate the tactile sensation of the legs of a ladybug touching the finger as it crawls. As real ladybugs crawl, they move alternately on three of their six legs^57^. By mimicking this motion, the cells in the tactile display were sequentially activated (Fig. 5c). Figure 5d shows a user testing environment that can deliver visual and tactile sensations simultaneously. The users wore the VR device and simultaneously placed their hands on a tactile display. Figure 5e shows a video of a ladybug crawling forward or backward (Movie S3 in the Supplementary information). Simultaneously, the PCM actuator of the tactile display was activated to simulate the walking motion of the ladybug (Fig. 5f). In the demonstration, five participants (all male, aged 27–34) wore the device on their fingertip while viewing a video of a ladybug performing a walking motion. Each participant experienced both a visual-only condition and a synchronized visual–tactile condition. After each condition, participants were asked to rate their perceived level of immersion using a 5-point Likert scale: (1) tactile feedback did not enhance immersion at all, (2) tactile feedback slightly did not enhance immersion, (3) neutral, (4) tactile feedback slightly enhanced immersion, and (5) tactile feedback strongly enhanced immersion. When only visual information was presented, participants reported recognizing the motion but without any accompanying physical sensation. In contrast, under synchronized visual–tactile stimulation, participants reported a crawling-like sensation on the fingertip that temporally matched the visual motion. The immersion scores for the synchronized condition were 4, 4, 5, 4, and 4 (mean = 4.2, standard deviation = 0.45), indicating that all participants perceived at least a slight enhancement of immersion, with one participant reporting a strong enhancement. These results suggest that synchronized tactile feedback can increase perceived immersion compared with visual feedback alone, consistent with previous findings on multisensory integration in VR and haptic perception^11^, although the small sample size limits statistical generalization.

## Conclusion

We developed a flexible tactile display based on PCM actuator arrays capable of delivering sophisticated tactile sensations. A thin, flexible device with a thickness of hundreds of micrometers was fabricated using a PI substrate and an elastic elastomer membrane, which could be wrapped around a fingertip for use as a flexible tactile display. The developed device could continuously achieve various displacements and forces by adjusting the driving power of the heater. Additionally, it operated faster than recently reported centimeter-scale liquid-to-gas PCM actuators (Table S2, Supplementary information), achieving actuating/releasing times of 370/600 ms and generating a maximum displacement of 580 µm with a low drive power of 300 mW. Even when attached to a curved surface with a radius of curvature of 10 mm, similar to that of a human finger, the device maintained a displacement deviation within 6.2% compared with that on a flat surface, thereby demonstrating stable actuation characteristics. When used with commercially available VR headsets, this tactile display enables the delivery of more immersive multisensory information.

## Materials and methods

### Fabrication of the microheater in PCM actuator

A PI precursor solution (polyamic acid, Merck) from Sigma-Aldrich was spin-coated onto a 4-in silicon wafer at 500 RPM for 60 s. A PI film with a thickness of 20 µm was then formed by curing sequentially at 80 °C for 1 h and at 250 °C for 2 h in a convection oven. Subsequently, the photoresist was patterned using photolithography, and Pt (20 nm) was deposited as an adhesive layer, followed by Au (100 nm) as the heater electrode, using a sputtering system (108 Auto, CRESSINGTON). After patterning the heater via a lift-off process, a passivation layer of PI was applied and cured using a spin-coating process (3000 RPM for 30 s, resulting in a thickness of 2 µm).

### Fabrication of PCM actuator arrays using the sublimation control system

We used the sublimation control setup developed for the formation of the PCM chamber. This setup includes an integrated Peltier device capable of controlling temperatures from –40 to 150 °C and a valve to adjust pressure and humidity (an image of the setup is provided in Fig. S1 of the Supplementary information). After placing the wafer with the patterned heater into the chamber, the temperature was lowered to –10 °C at atmospheric pressure, causing water vapor in the air to sublimate into the ice on the surface of the device. Subsequently, an uncured elastomer (Ecoflex^TM^ 00-35 Fast, Smooth-On) was spin-coated at 1000 RPM for 30 s and cured at room temperature for 10 min. After curing, when the heater was activated, heating occurred only in the heated area, causing the water droplets on the Ecoflex membrane to vaporize. This resulted in delamination between the Ecoflex and PI layers only in the heater area, thus forming a PCM chamber.

### Characterization of the PCM actuators

The surface temperature of the PCM actuator during the heater operation was measured using a thermal imaging camera (E86, FLIR Systems). A laser displacement sensor (LJ-X8060, KEYENCE) was used to measure the driving displacement in real time, and power was applied through a power supply (EDU36311A, Keysight) to drive the heater. In addition, a thin layer of graphite (Graphit 33, KONTAKT CHEMIE) was sprayed to accurately measure the displacement of the transparent membrane. To measure the driving force, the device was placed on a stage with z-axis movement capability, and an Ecoflex layer with skin-like strength was placed in contact with the integrated load cell. The driving force was measured in real time using a force gauge (DTG-1, Digitech).

### Visual and tactile interface demonstration

To deliver realistic information to users in a VR environment, we present a proof-of-concept multimodal VR device that integrates a commercial VR headset with a PCM actuator-based tactile display to transmit visual information. A video of a ladybug crawling was created (After Effects, Adobe), and the tactile display was synchronized to the steps of the ladybug to deliver the tactile sensation of its movements. During the operation, only specific cells were activated for the desired duration using an Arduino MCU (Mega 2560, Arduino) and transistor arrays (IRFZ44N, Infineon). Informed consent was obtained from all the participants who volunteered to participate in these studies. All testing reports conformed to the ethical requirements of Yonsei University.

## Supplementary information

Movie1_actuator operation

Movie2_force measurement

Movie3_crawling

Supplemental Material File #1
