# Supplementary material for: Flexible microscale tactile display with liquid-to-gas phase-change actuator array
Source: Microsyst Nanoeng. 2026 May 19;12:185. doi: 10.1038/s41378-026-01288-z (PMC13187177; doi:10.1038/s41378-026-01288-z)
Supplement: Supplementary file 4 — Supplemental Material File #1 [file 41378_2026_1288_MOESM4_ESM.docx]

**Supporting Information**

**Flexible microscale tactile display with liquid-to-gas phase-change actuator array**

Sangjun Sim^1, ‡^, Kyubin Bae^2, ‡^, Kyuhyun Hwang^1^, Seunghwan Koh^1^, and Jongbaeg Kim^1,^ *

^1^School of Mechanical Engineering, Yonsei University

50 Yonsei-ro, Seodaemun-gu, Seoul 03722, Republic of Korea

^2^Walker Department of Mechanical Engineering, The University of Texas at Austin,

Austin, TX, 78712, USA

Corresponding Author

Prof. Jongbaeg Kim^*^

School of Mechanical Engineering, Yonsei University

E-mail: [kimjb@yonsei.ac.kr](mailto:kimjb@yonsei.ac.kr)

Author Contributions

‡These authors equally contributed to this work.

**KEYWORDS**: phase-change materials, microactuator, tactile display, flexibility, multisensory virtual reality device

**Contents:**

1. Analytical model for liquid-to-gas phase change actuation (Note 1)

2. Optical image of the sublimation setup for creating ice particles under controlled humidity, pressure, and temperature conditions (Fig. S1)

3. Fabrication process of the microscale tactile display, showing detailed steps including microheater patterning and Ecoflex chamber forming (Fig. S2)

4. Time-lapse images of water droplets vaporizing upon heater activation, forming an Ecoflex chamber localized to the heater area (Fig. S3)

5. Optical images of various tactile display sizes and their flexibility on a fingertip (Fig. S4)

6. Optical images of the fabricated tactile display on a concave surface, showing selective cell activation (Fig. S5)

7. Characterization of the operating temperature of the microheater (Fig. S6)

8. Thermal characterization of neighboring-cell interference during actuation (Fig. S7)

9. Schematic of the characterization setup of the tactile display with equipment for imaging, displacement measurement, and thermal analysis (Fig. S8)

10. Quantitative validation of the phase-change actuation model (Fig. S9)

11. Comparison of performance between recently reported flexible tactile displays and our device (Table S1)

12. Comparison of performance between recently reported PCM actuators and our device (Table S2)

13. Environmental stability of the PCM actuator under varying humidity and temperature conditions (Fig. S10)

14. Long-term stability of the water-based PCM actuator after one year of storage at room temperature (Fig. S11)

15. Optical microscope images of the Ecoflex membrane adhered to the PI substrate (Fig. S12)

16. Setup for force measurement with a pressure gauge and push-pull stand (Fig. S13)

17. Repetitive force measurement of the tactile display over 60 cycles at 50 mW (Fig. S14)

18. Force output of 15 cells of tactile display under varying power levels (Fig. S15)

19. Operation of the PCM actuator (Movie S1 and S2)

20. Visual and tactile information showing a virtual ladybug crawling (Movie S3)

**Supporting Note 1. Analytical model for liquid-to-gas phase change actuation**

To quantitatively rationalize this experimentally observed transition in the power–displacement slope, we have developed and added an analytical framework that links the input electrical power to actuator displacement through a sequence of thermodynamic and mechanical relations. The proposed model captures the dominant physics of the liquid-to-gas phase-change actuation and explains the slope change observed in the vaporization regime.

Specifically, the analytical model consists of three coupled stages:

(1) energy balance governing liquid–gas phase transition,

(2) pressure generation within the geometrically constrained PCM chamber, and

(3) pressure-induced deformation of the elastomer membrane.

**(1) Energy balance and vapor generation**

When electrical power is applied to the microheater, the supplied energy is transferred to the PCM with an effective thermal efficiency $\eta$. In the high-power regime, where liquid-gas phase transition dominates, the majority of the input energy contributes to vapor generation rather than sensible heating. This process can be described by a simple energy balance:

$$P\cdot\eta=\dot{m}L_{v}$$

where $P$ is the input electrical power, $\eta$ is the effective thermal efficiency from heater to PCM, $\dot{m}$ is the mass generation rate of vapor, and $L_{v}$ is the latent heat of vaporization of water.

**(2) Vapor generation to internal pressure rise**

Because the PCM chamber is mechanically confined by the elastomer and the substrate, the generated vapor cannot freely expand. Instead, the increase in vapor mass leads to a rise in internal pressure. Assuming ideal-gas behavior for the water vapor, the internal pressure $P_{\text{int}}$ is given by:

$$P_{\text{int}}=\frac{nRT}{V_{\text{eff}}}$$

Where $n$ is the number of moles of vapor, $R$ is the universal gas constant, $T$ is the absolute temperature, and $V_{\text{eff}}$ is the effective chamber volume.

Using $n=m/M$, where $M$ is the molar mass of water, the incremental pressure increase associated with vaporization becomes:

$$\Delta P_{\text{int}}=\frac{RT}{V_{\text{eff}}ML_{v}}\cdot\eta P$$

This expression shows that, once vaporization begins, the internal pressure increases linearly with the applied power due to the constrained chamber volume.

**(3) Pressure-induced membrane deformation**

The generated internal pressure is converted into mechanical displacement through deformation of the elastomer membrane. The membrane can be approximated as a clamped circular membrane with radius $a$ and bending rigidity $D$, where:

$$D=\frac{Eh^{3}}{12\left( 1-\nu^{2} \right)}$$

With $E$, $h$, $\nu$ denoting the Young’s modulus, thickness, and Poisson’s ratio of the elastomer, respectively.

Under uniform internal pressure, the central displacement δ of the membrane in the small-deformation regime can be expressed as:

$$\delta=\frac{P_{\text{int}}a^{4}}{64D}$$

This relation indicates that membrane displacement scales linearly with internal pressure, and thus with the generated vapor mass.

**(4) Resulting power-displacement relationship**

By combining the above relations, the actuator displacement as a function of applied power in the vaporization regime can be written as:

$$\delta\left( P \right)=\left( \frac{RTa^{4}}{64DV_{\text{eff}}ML_{v}} \right)\cdot\eta P$$

This analytical expression predicts a linear power–displacement relationship, consistent with the experimental data in Fig. 3c. Importantly, the transition from a shallow slope at low power to a steeper slope at higher power naturally emerges from the model, reflecting the transition from sensible heating to latent-heat-dominated vaporization.

Using representative geometric and material parameters of the fabricated device (membrane radius, thickness, and elastic modulus), the predicted displacement-per-power scaling agrees with the experimentally measured slope (≈2.9 µm$\cdot$mW^–1^) within the same order of magnitude, thereby providing analytical validation of the proposed actuation mechanism.

**
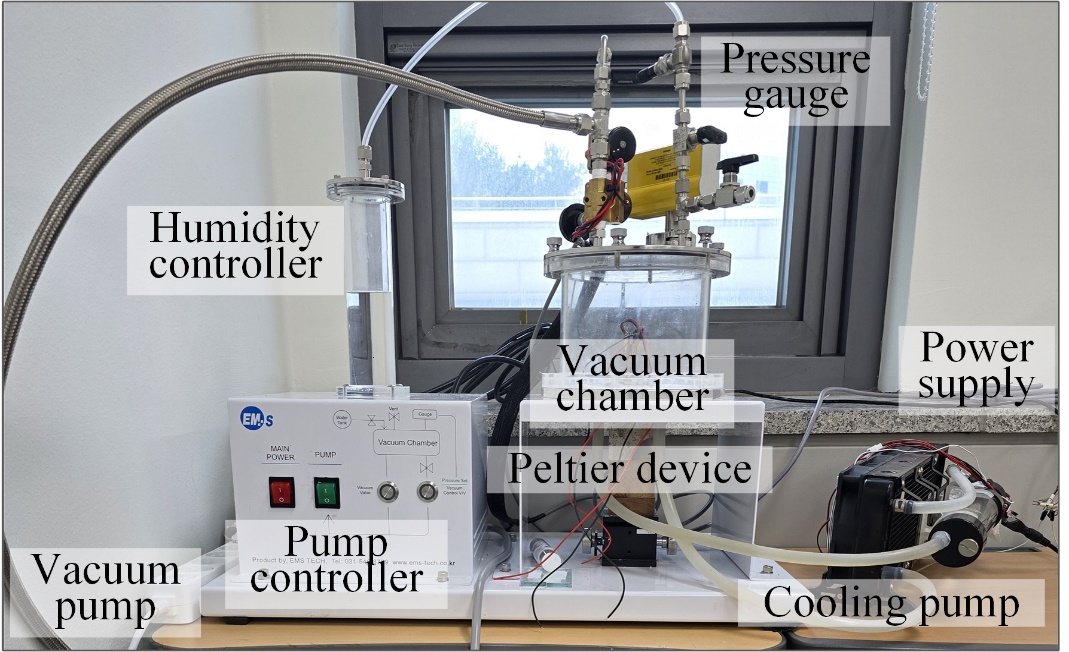
**

**Fig. S1.** Optical image of the sublimation setup used for creating ice particles during device fabrication. The setup includes a humidity controller and pressure gauge for regulating humidity and pressure within the vacuum chamber. A Peltier device controls the temperature, with a cooling pump employed to dissipate heat from the hot side of the Peltier element, enhancing its cooling efficiency. Additional components include a vacuum pump and pump controller, enabling precise environmental control necessary for ice particle formation.


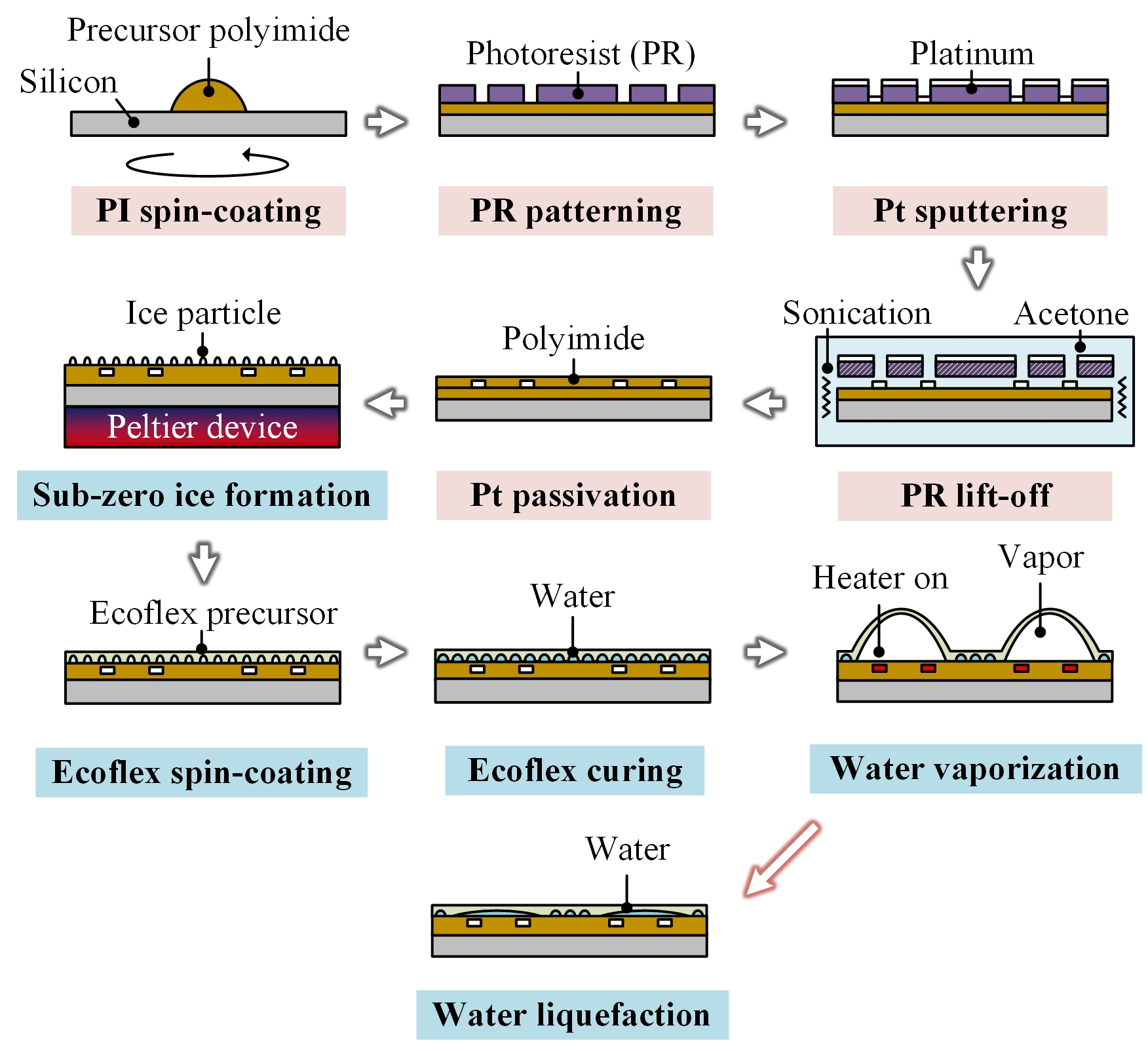


**Fig. S2.** Fabrication process of the microscale tactile display. (ⅰ) Polyimide precursor is spin-coated onto a silicon substrate, followed by photoresist (PR) patterning and platinum sputtering. (ⅱ) PR liftoff is performed using sonication in an acetone bath, leaving the patterned Pt layer, which is subsequently passivated with polyimide. (ⅲ) Ice particles are formed on the surface using a Peltier device at sub-zero temperatures. (ⅳ) Ecoflex precursor is spin-coated onto the ice-decorated surface at a low temperature to prevent ice liquefaction. (ⅴ) During Ecoflex curing, the ice melts into water. Finally, heater activation induces water vaporization, and upon deactivating the heater, the water re-liquefies, completing the batch fabrication of the tactile display.


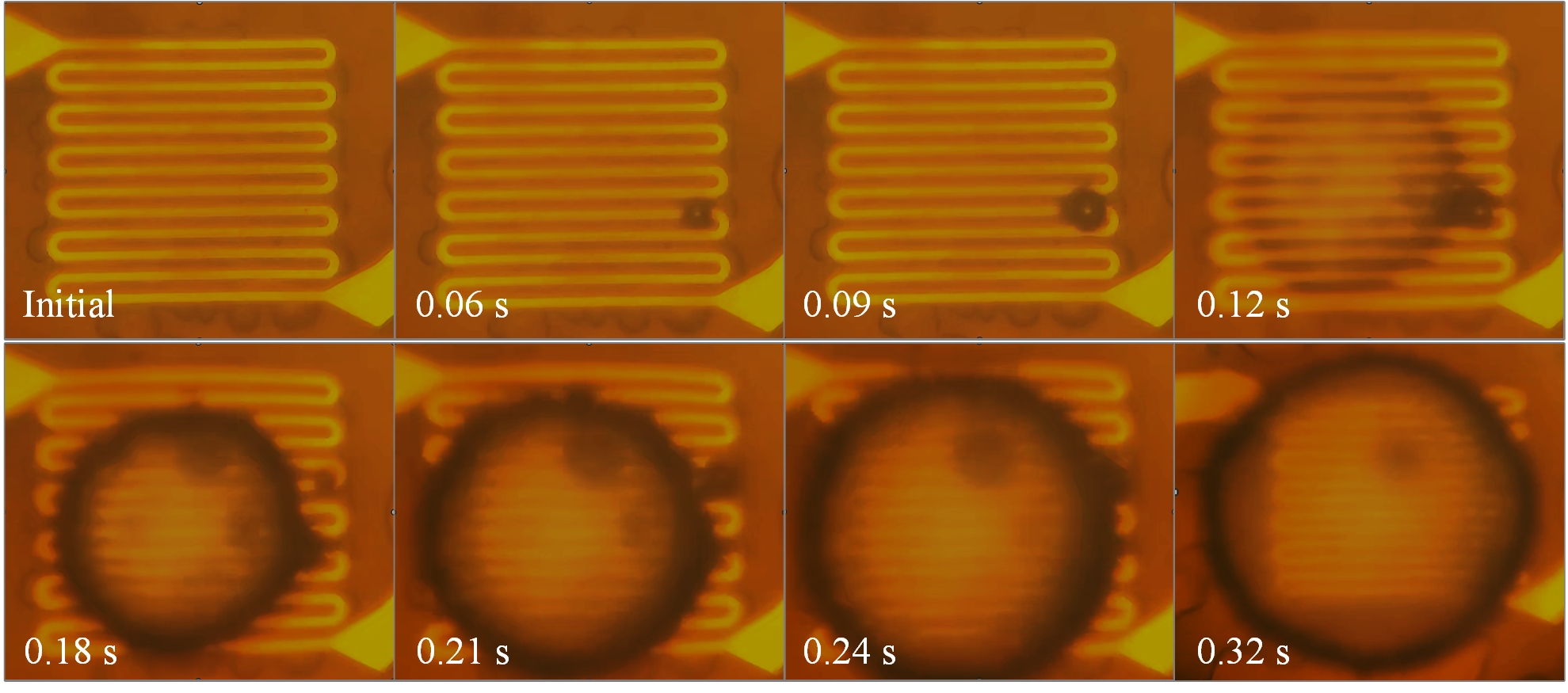


**Fig. S3.** Time-lapse optical images showing the vaporization of water droplets and the formation of an Ecoflex chamber over the heated area. The images illustrate the dynamic process where water droplets evaporate upon heater activation, leading to the confinement of the Ecoflex chamber within the heater region. Time intervals are labeled in each image to indicate the progression of chamber formation.

**
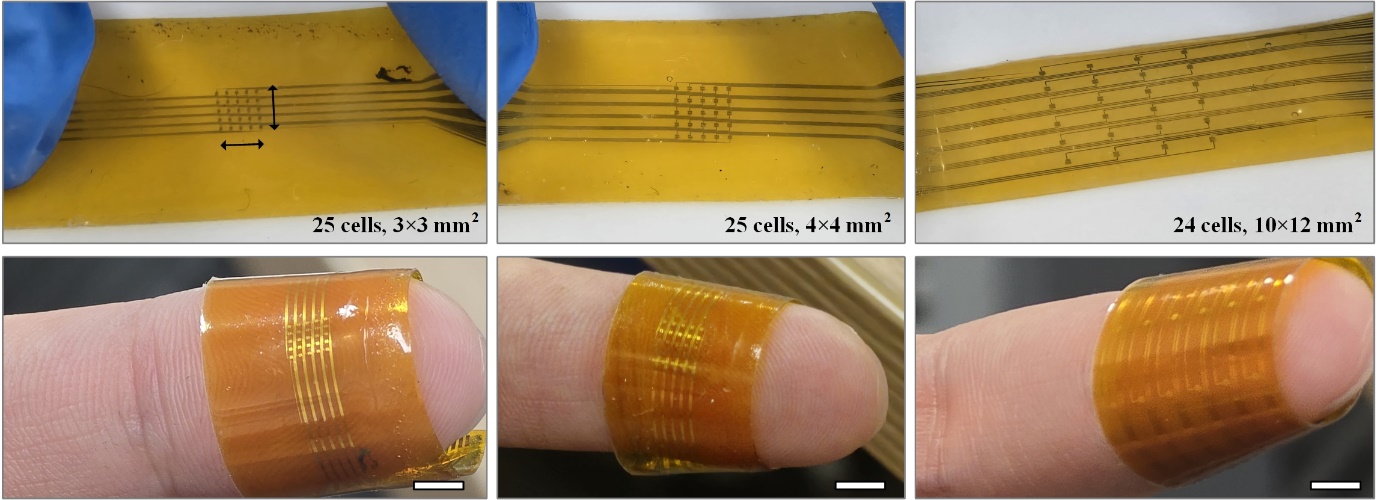
**

**Fig. S4. Optical images of the fabricated tactile display with various cell array sizes (25 cells in a 3 × 3 mm² area, 25 cells in a 4 × 4 mm² area, and 24 cells in a 10 × 12 mm² area) and its application on a fingertip. The images show the tactile display devices wrapped around a fingertip, demonstrating their flexibility and conformability to curved surfaces (scale bars: 2 mm).**

**
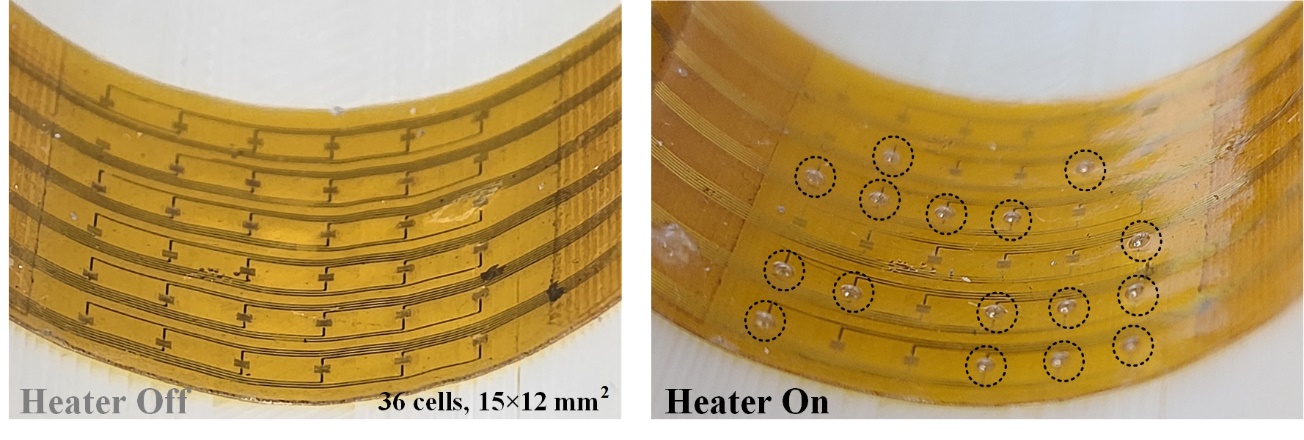
**

**Fig. S5.** Optical images of the fabricated tactile display with 36 cells (15 × 12 mm² area) before and after heater activation. The display is mounted on a concave surface to demonstrate flexibility. Before activation, all the cells remain inactive. After activation, specific cells (circled) are selectively actuated, highlighting the ability of the device to conform to curved surfaces while providing localized tactile feedback.

**
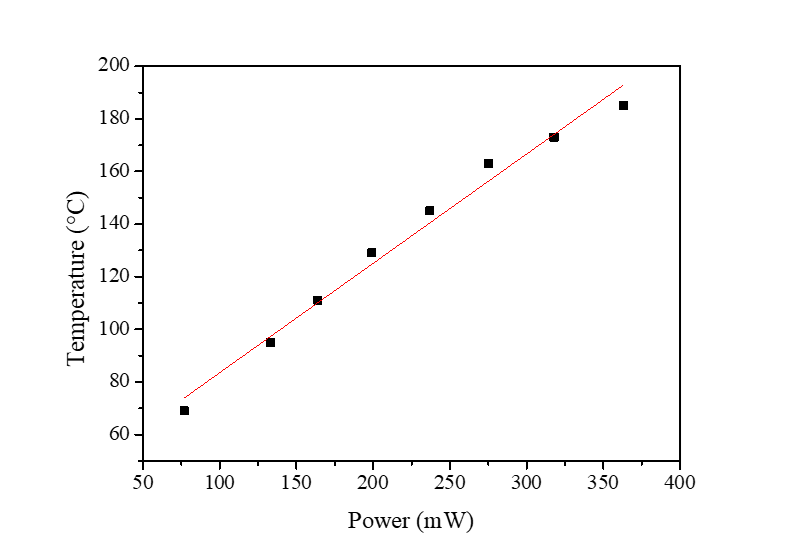
**

**Fig. S6.** Characterization of the temperature of the flexible microheater under various applied power levels ranging from 77 to 363 mW.
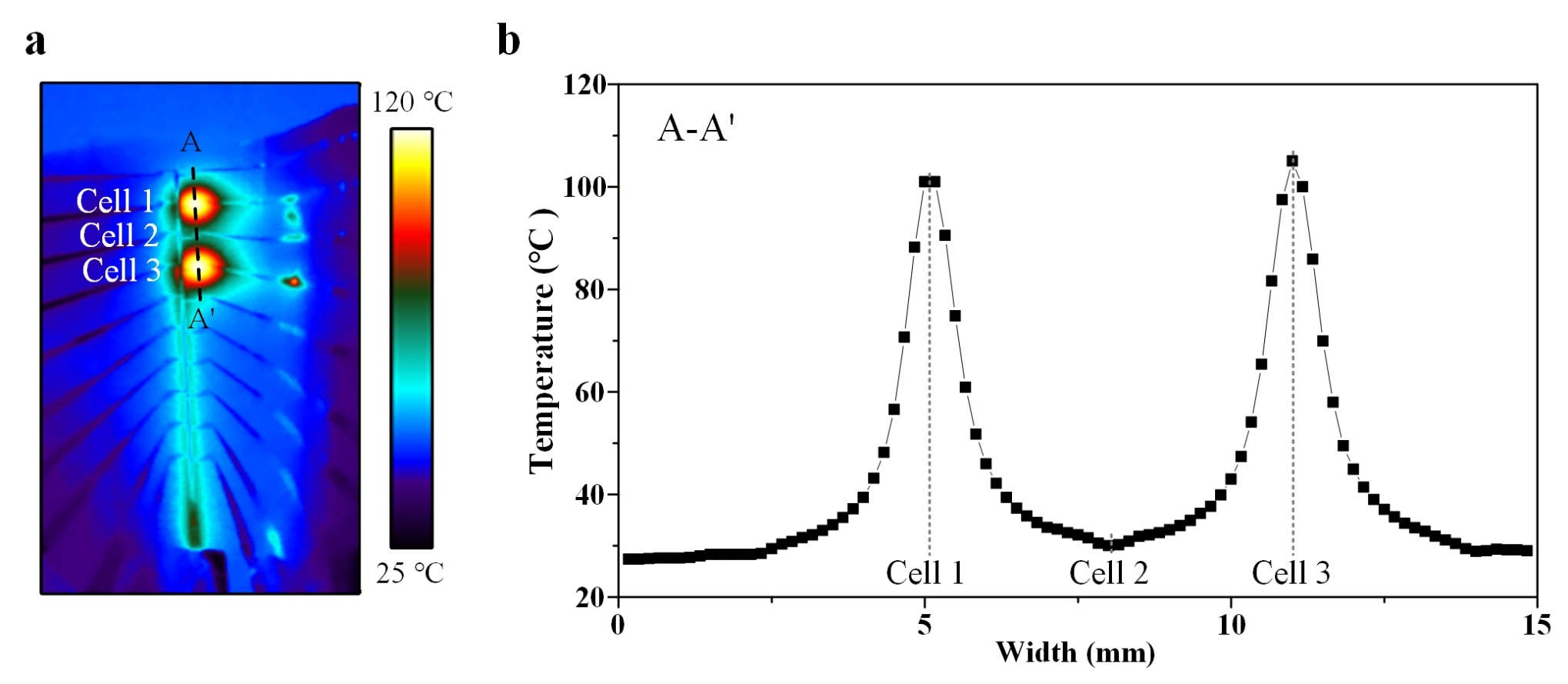


**Fig. S7.** Thermal characterization of neighboring-cell interference during actuation. (a) Infrared thermal image of the actuator array with Cells 1 and 3 activated, showing localized heating. The high-temperature region (>100 °C) is confined to approximately 1 mm around each heater. (b) Temperature profile along the A–A′ line, demonstrating rapid thermal decay outside the heated zone. Given the array pitch of 3 mm, which exceeds the measured thermal spread, adjacent cells experience negligible thermal interference, with the inter-cell region maintaining a temperature of approximately 30 °C even under heater surface temperatures exceeding 100 °C.

**
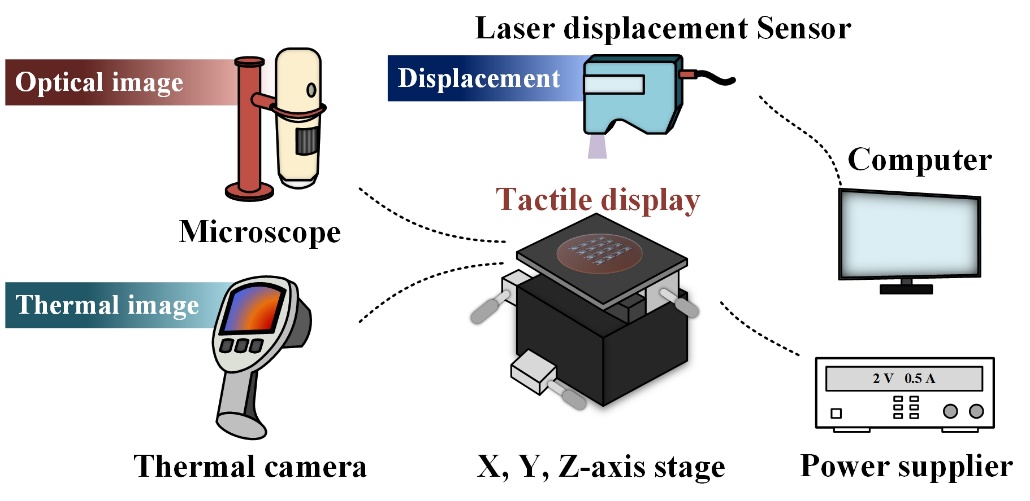
**

**Fig. S8.** Schematic of the measurement setup used for characterizing the tactile display. The setup includes a microscope for capturing optical images, a laser displacement sensor for measuring displacement, and a computer for monitoring data. A power supply is used to apply voltage to the tactile display, which is mounted on an X-, Y-, and Z-axis stage for precise positioning. A thermal camera measures the generated heat, allowing the thermal analysis of the device during operation.

**
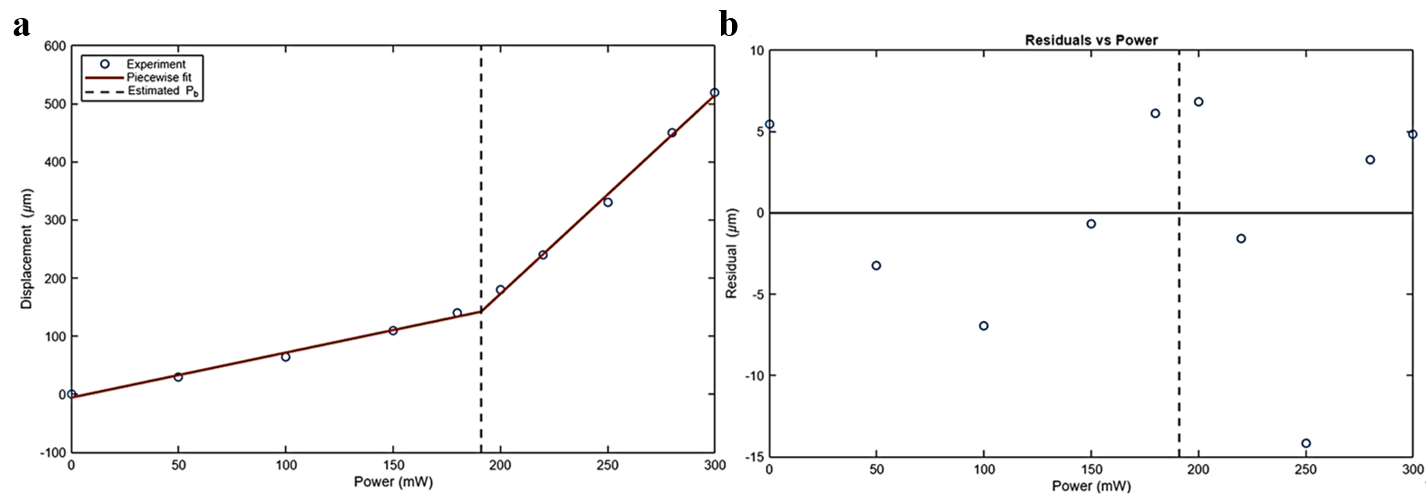
**

**Fig. S9. Quantitative validation of the phase-change actuation model.** (a) Complete piecewise-linear fitting of the power–displacement data, showing the data-driven estimation of the threshold power. (b) Residuals versus power, demonstrating good agreement between the model and experiment across most of the power range.

**Table S1.** Comparison of performance between recently reported tactile displays and our device.

| **Working mechanism** | **Size [mm]** | | **Displacement [mm]** | **Normalized displacement [mm/mm]** | **Operating power [W]** | **Flexibility** | **Ref.** |
| --- | --- | --- | --- | --- | --- | --- | --- |
| **Phase change** | | **0.5** | **0.58** | **1.16** | **0.3** | **O** | **Our work** |
| Phase change | | 6 | 1.93 | 0.322 | 0.65 | O | [45] |
| Phase change | | 9 | 4 | 0.445 | 0.1 | O | [51] |
| Dielectric elastomer actuation | | 10 | 1 | 0.1 | 0.1 | O | [48] |
| *ERM motor | | 12 | 0.035 | 0.0029 | 0.00175 | O | [10] |
| *ERM motor | | 7 | 0.23 | 0.0329 | 0.158 | O | [47] |
| Pneumatic | | 7 | 5 | 0.714 | 1.8 | X | [49] |
| Combustion | | 5 | 6.25 | 1.25 | 2.9 | O | [50] |

*** ERM: Eccentric rotating mass**

* The normalized displacement is defined as the actuator displacement divided by its diameter.

**Table S2.** Comparison of performance between recently reported PCM actuators and our device.

| **Working Fluid** | **Size [mm]** | **Displacement [mm]** | **Operating power [W]** | **Response time (on/off) [s]** | **Ref.** |
| --- | --- | --- | --- | --- | --- |
| Water | 0.5 | 0.58 | **0.3** | **0.37/0.6** | **Our work** |
| Ethanol | >10 | 14.6 | 600 | 90 | [32] |
| Novec 7000 | 21 | 18 | 1.5 | 15/30 | [44] |
| N/A | 6 | 1.93 | 0.65 | 2 | [45] |
| Methanol | 15 | N/A | N/A | 180 | [46] |
| Ethanol | 20 | 80% | 9 | 22 | [S1] |
| Novec 7000 | 100 | 16.5 | 15 | >10 | [S2] |
| Novec 7000 | 21.8 | 17 | 3.5 | 246 | [S3] |
| Novec 7000 | 13 | N/A | 1.8 | 20 | [S4] |
| Novec 7100 | 23 | 32 | 1.6 | 178 | [S5] |
| Ethanol | 24 | 18.7 | N/A  (Driving voltage = 5 V) | 20 | [S6] |

S1. Bilodeau, R. A. *et al.* *2018 IEEE International Conference on Soft Robotics (RoboSoft)* 288–294 (2018).

S2. Narumi, K. *et al.* *IEEE Robot. Autom. Lett.* 5, 3915 (2020).

S3. Ueno, S. & Monnai, Y. *IEEE Robot. Autom. Lett.* 5, 6483 (2020).

S4. Uramune, R., Maeda, T., Kawahara, Y. & Narumi, K. *IEEE Access* 10, 16830 (2022).

S5. Yoon, Y. *et al.* *Chem. Eng. J.* 451, 139217 (2023).

S6. Wei, F., Zhai, Z. & Yang, L. *ACS Appl. Electron. Mater.* **5**, 5527 (2023).


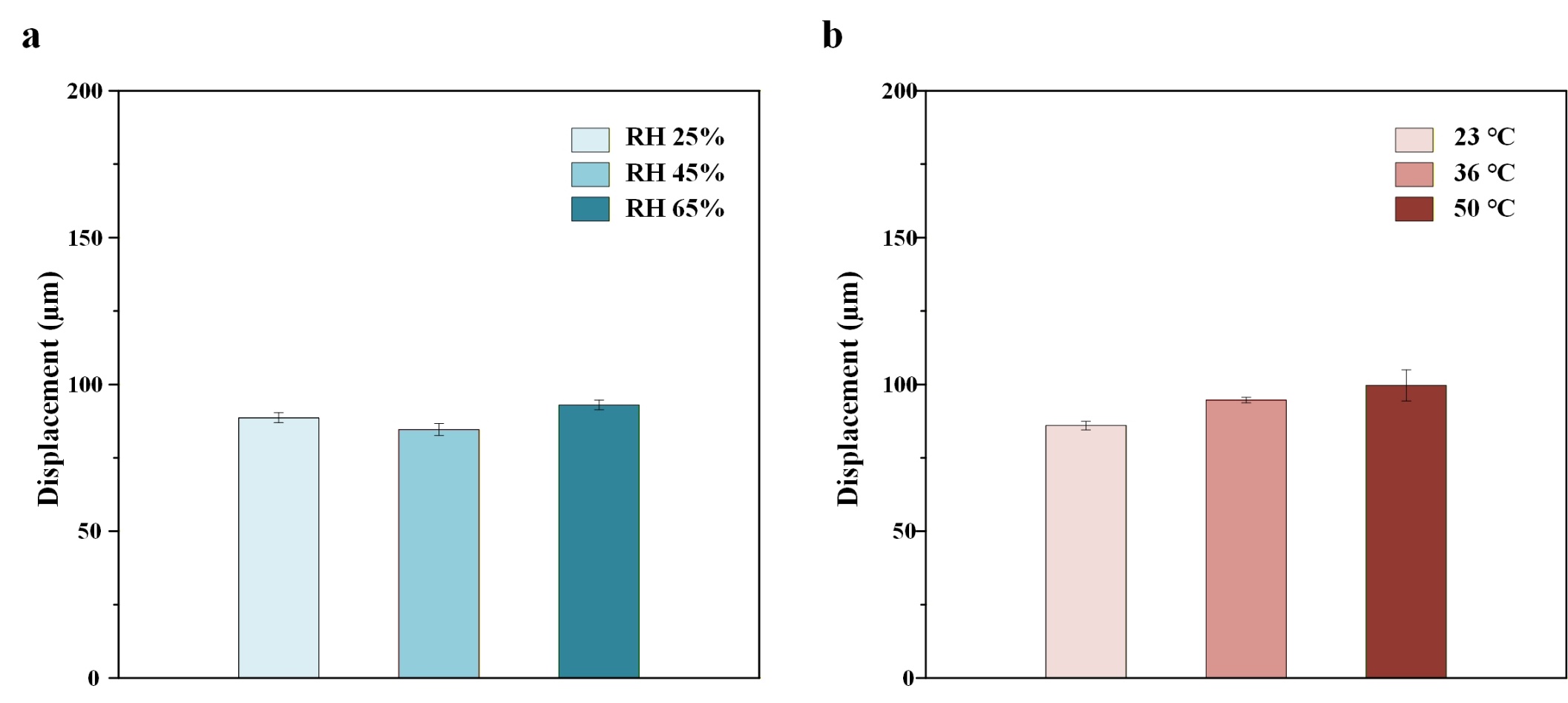


**Fig. S10.** Environmental stability of the PCM actuator under varying humidity and temperature conditions. (a) Displacement measured at relative humidities of 25%, 45%, and 65%, showing variations within 9%, indicating minimal influence of humidity on actuation performance. (b) Displacement measured at ambient temperatures of 23 °C, 36 °C, and 50 °C. A moderate increase of approximately 13.8% is observed as the temperature rises from 23 °C to 50 °C due to facilitated phase transition at higher temperatures. Overall, the actuator maintains stable and consistent behavior across different environmental conditions.


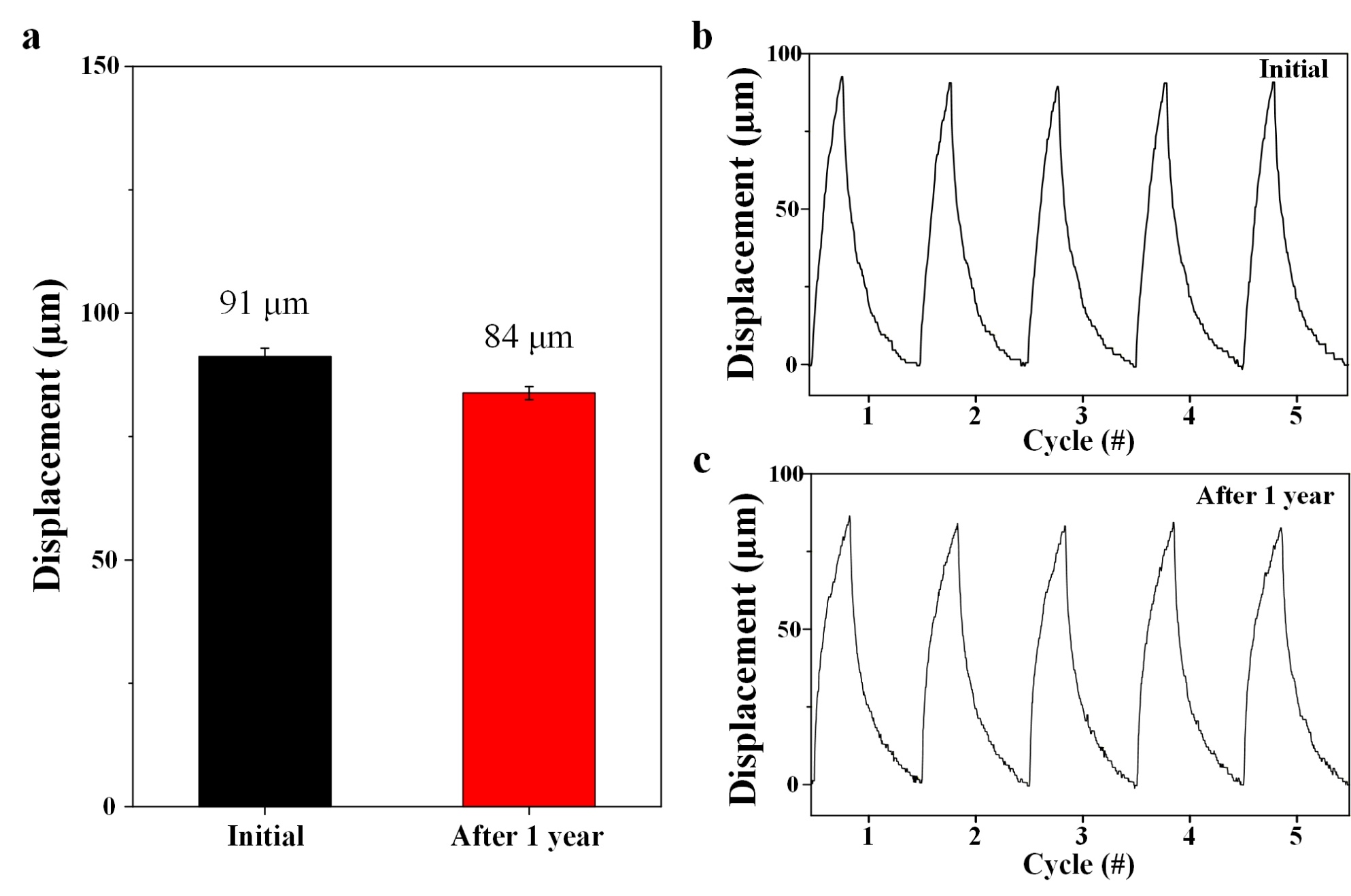


**Fig. S11.** Long-term stability of the water-based PCM actuator after one year of storage at room temperature. (a) Comparison of peak displacement measured initially (91 µm) and after 1 year (84 µm), showing minimal performance degradation. (b) Displacement profiles of five consecutive actuation cycles in the initial state. (c) Displacement profiles of five consecutive actuation cycles after 1 year, demonstrating consistent temporal behavior and stable actuation characteristics over extended storage.


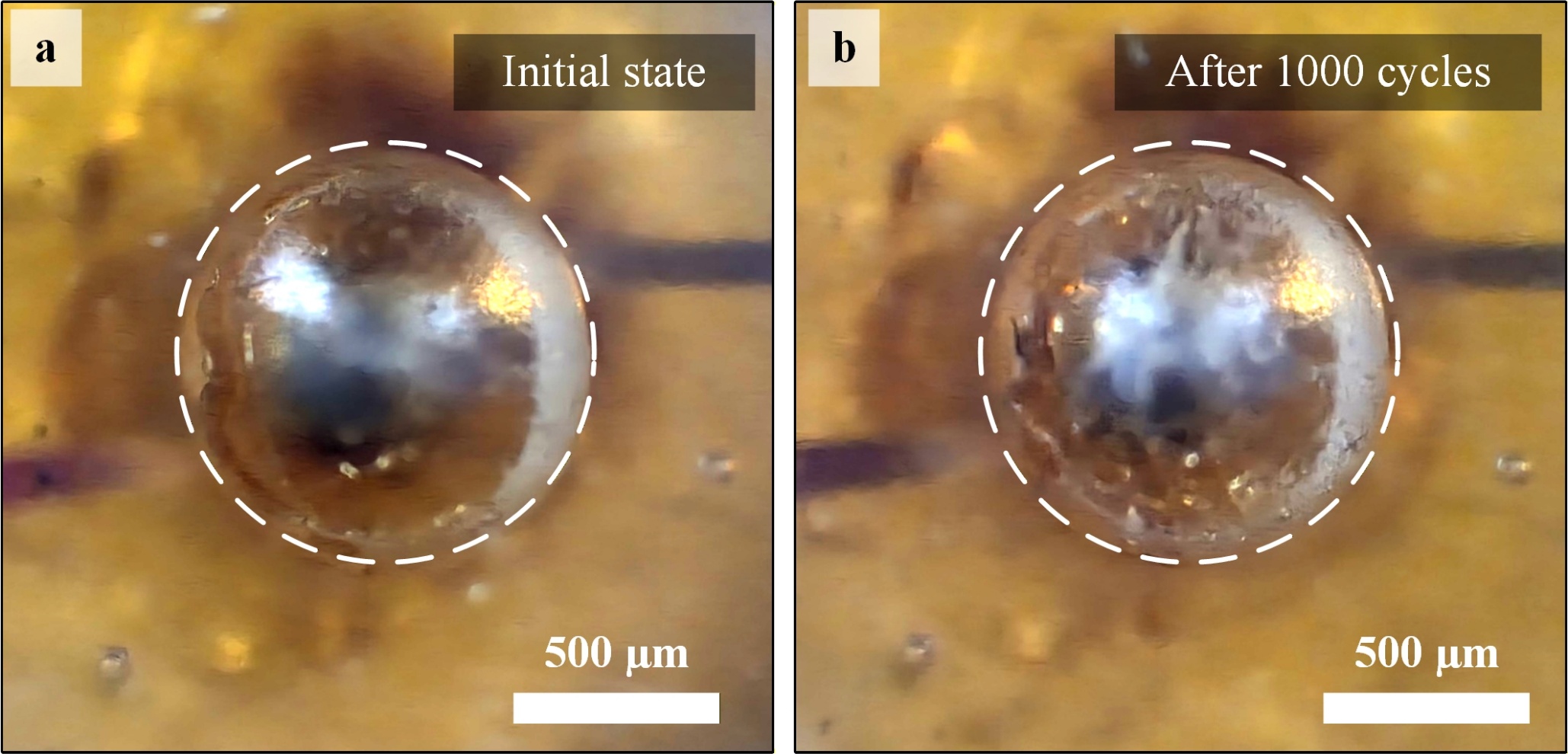


**Fig. S12.** Optical microscope images of the Ecoflex membrane adhered to the PI substrate (a) in the initial state and (b) after 1000 actuation cycles. No delamination or peeling is observed, indicating stable adhesion and mechanical integrity of the Ecoflex–PI interface under repeated phase-change actuation.


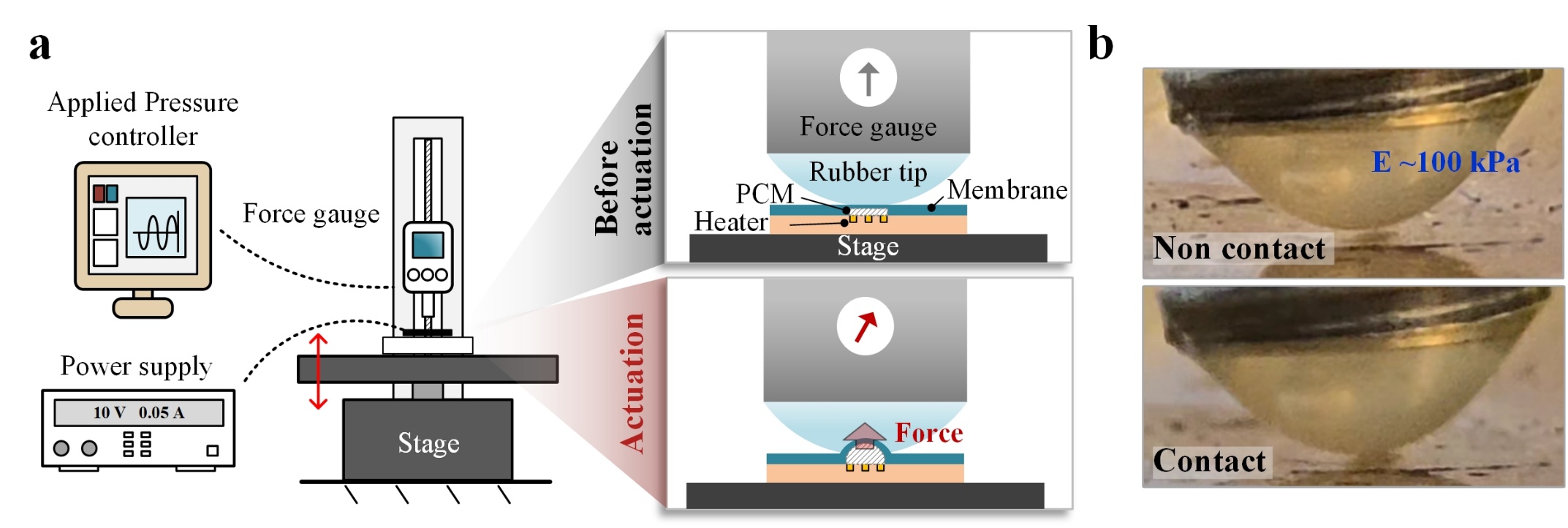


**Fig. S13.** (a) Setup for the force measurement of the tactile display, including an applied pressure controller, a force gauge, and a power supply. The tactile display is positioned on a stage, and the force gauge is equipped with a rubber tip (Young’s modulus: ~100 kPa) coated with a polymer that mimics the stiffness of human skin to simulate realistic contact conditions. (b) Enlarged optical images of the contact interface between the pressure gauge tip and the tactile display. Before actuation, a small gap remains between the tip and the display, resulting in a noncontact state. Upon the actuation of the tactile display, contact is achieved, and the device exhibits force.

**
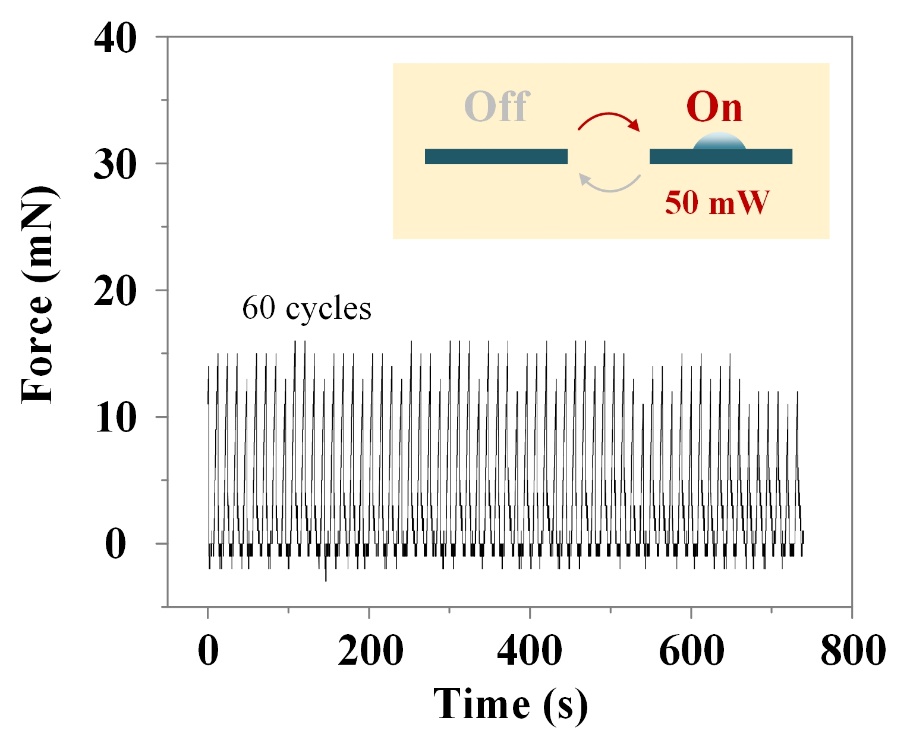
**

**Fig. S14.** Force measurement of the tactile display over 60 actuation cycles at an input power of 50 mW. The device consistently generates a similar force of approximately 15 mN throughout 60 cycles, demonstrating stable performance under repeated actuation.


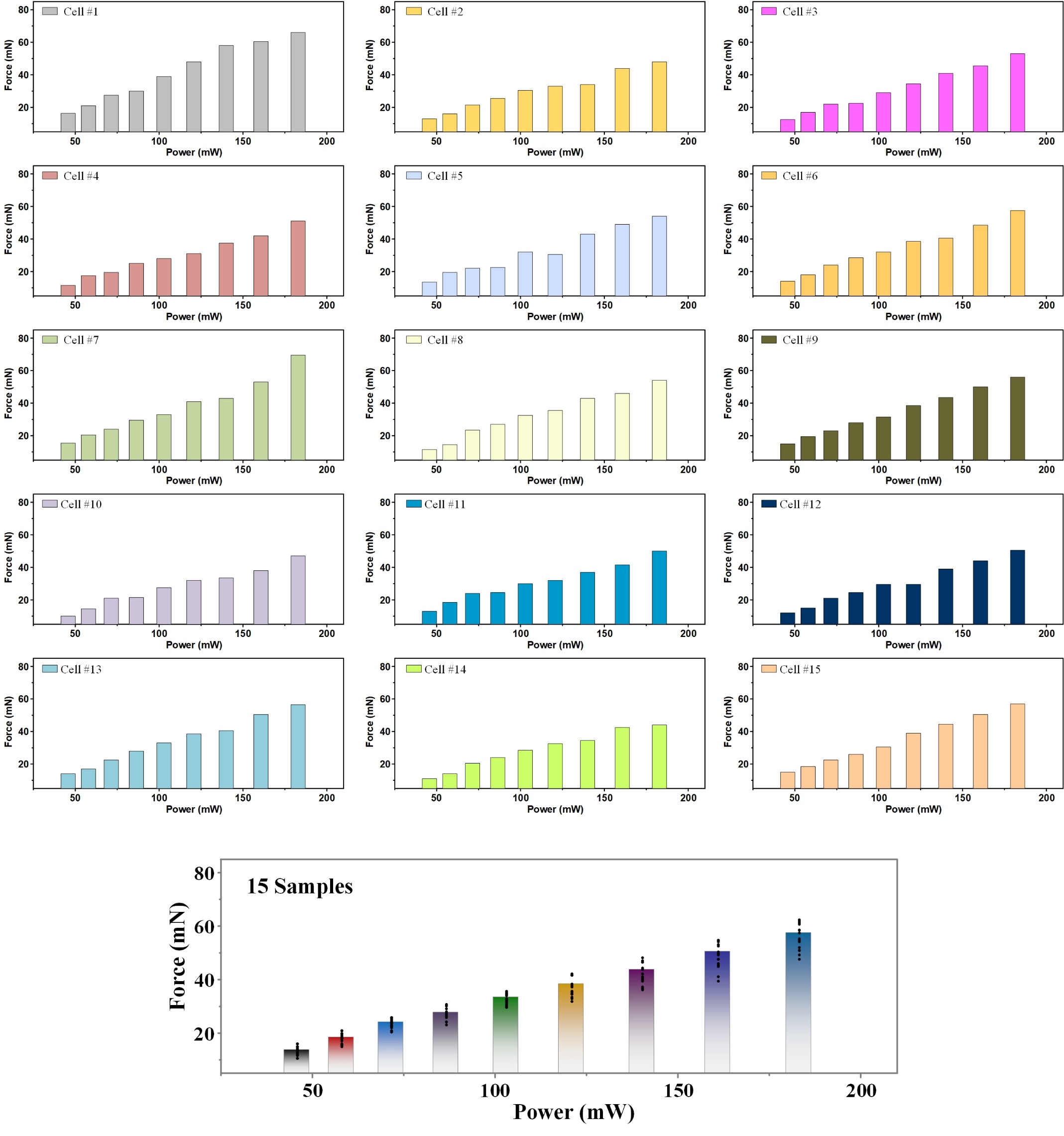


**Fig. S15.** Force generated by each of the 15 tactile display cells as a function of the applied power, measured at power levels ranging from 40 to 180 mW. Each subplot shows the force response of an individual cell, illustrating a consistent increase in force with higher power input. The bottom plot presents the average force across all 15 cells at each power level, demonstrating the uniform performance of the cells without significant variation.
